# Lipopolysaccharide enhances ADAR2 which drives Hirschsprung's disease by impairing miR‐142‐3p biogenesis

**DOI:** 10.1111/jcmm.13652

**Published:** 2018-06-29

**Authors:** Lei Peng, Hua Zhang, Yang Su, Qiyang Shen, Chunxia Du, Hua Xie, Hongxing Li, Jin Yan, Ziyang Shen, Weiwei Jiang, Yankai Xia, Xiaoqun Xu, Weibing Tang

**Affiliations:** ^1^ Department of Gastroenterology First Affiliated Hospital of Nanjing Medical University Nanjing China; ^2^ Department of Pediatric Surgery Children's Hospital of Nanjing Medical University Nanjing China; ^3^ Ministry of Education Key Laboratory of Modern Toxicology Nanjing Medical University Nanjing China; ^4^ Department of Hepatobiliary & Pancreatic Surgery The Affiliated Huaian No.1 Peoples Hospital of Nanjing Medical University Huaian China; ^5^ State Key Laboratory of Reproductive Medicine Institute of Toxicology School of Public Health Nanjing Medical University Nanjing China

**Keywords:** A‐to‐I editing, Hirschsprung's disease, lipopolysaccharide, migration, proliferation

## Abstract

Researches over the past decade suggest that lipopolysaccharide is a dominant driver of gastrointestinal motility and could damage the enteric neuron of rat or porcine. However, it remains poorly defined whether LPS participates in Hirschsprung's disease (HSCR). Here, we discovered that LPS increased in HSCR tissues. Furthermore, LPS treatment suppressed the proliferation and differentiation of neural precursor cells (NPCs) or proliferation and migration of human 293T cells. ADAR2 (adenosine deaminase acting on RNA2)‐mediated post‐transcriptional adenosine‐to‐inosine RNA editing promotes cancer progression. We show that increased LPS activates ADAR2 and subsequently regulates the A‐to‐I RNA editing which suppresses the miR‐142 expression. RNA sequencing combined with qRT‐PCR suggested that ADAR2 restrain cell migration and proliferation via pri‐miR‐142 editing and STAU1 up‐regulation. In conclusion, the findings illustrate that LPS participates in HSCR through the LPS‐ADAR2‐miR‐142‐STAU1 axis.

## 1 INTRODUCTION

Hirschsprung's disease (HSCR) is a rare congenital disease characterized by the absence of ganglion cells.^1^ The incidence of HSCR in live births is 1:5000, and the sex ratio of affected baby boys and girls is 4:1.^2^ HSCR is a complex disease caused by gene‐environment interaction. Genetic studies have found that some protein coding genes including RET, EDNRB^3^ and non‐protein coding genes such as miR‐218‐1,^4^ LOC101926975^5^ associated with HSCR. However, the environmental factors contributing to HSCR are still insufficiently investigated,just BP‐3^6^ is revealed to be related to HSCR.

Lipopolysaccharide (LPS), a microbiota‐associated factor belonging to by‐products of microbial metabolism and microbial cell composition, is a primary constituent of the Gram‐negative bacterial membrane^7^ that influences gastrointestinal motility.^8^ Previously, in vitro LPS exposure has led to loss of porcine and rat intestinal neuron.^9^, ^10^ Furthermore, LPS has been demonstrated dramatically to enhance the editing activity of ADAR1.^11^

Accumulated evidence suggests that adenosine deaminase acting on RNA (ADAR) enzymes, such as ADAR1 and ADAR2, accelerates tumour development.^12^, ^13^ The ADAR enzyme is a double‐stranded RNA (dsRNA) binding protein that regulates adenosine into inosine (A to I) at the post‐transcriptional level.^14^ ADAR2, an indispensable enzyme for brain development and function,^15^ is enough to inhibit glioblastoma proliferation or cancer progress.^16^ Then, we speculated that ADAR2 could be associated with the development of the enteric nervous system (ENS). Recently, it has been shown that ADARs may edit miRNA precursors, thereby altering its maturation step.^17^

Here, we demonstrated that increased concentration of LPS enhanced the activity of ADAR2. Then, ADAR2‐mediated A‐to‐I editing suppressed the development of ENS by impairing miR‐142‐3p biogenesis, resulting in unopposed STAU1 expression. The experiments were conducted to unravel the biological roles of LPS and underlying regulative mechanism between LPS‐ADAR2‐miR‐142‐3p‐STAU1, which may contribute to the pathogenesis of HSCR.

## 2 MATERIALS AND METHODS

### 2.1 Clinical tissue samples

The Nanjing Medical University Ethics Committee approved the study. HSCR and control tissues were obtained from Nanjing Children's Hospital from 2011 to 2016 and then stored at −80°C. In this study, control samples refer to samples confirmed no HSCR or other intestinal nerve malformations. HSCR sample was confirmed by examination of intestinal ganglia. The clinical data of the patients are summarized in Table 1.

**Table 1:** Demographic clinical features of study subjects

Variable	Control (n = 48)	HSCR (n = 48)	*P*
Age (d, mean, SE)	127.3 (8.105)	124.1 (9.542)	.8034^a^
Weight (kg, mean, SE)	5.848 (0.151)	5.834 (0.188)	.9539^a^
Sex (%)			
Male	41 (85.41)	38 (79.17)	.59^b^
Female	7 (14.59)	10 (20.83)	

a Student's *t* test.

b Two‐sided chi‐squared test.

### 2.2 Neural precursor cell culture

The Institute of Cancer Research (ICR) E16.5 mice were killed by cervical dislocation. Then, we separate the outer layer of the intestinal tract under a stereoscopic microscope. The remaining tissue was triturated to 0.5‐1 mm^3^ and incubated 60 minutes in 1 mg/mL collagenase. Then, the small pieces were centrifuged and filtered through a 200‐μm filter to produce a single‐cell suspension using DMEM‐F12 supplemented with N2 (1%), B27 (2.0%), bFGF (20 ng/mL), EGF (20 ng/mL). The cells were placed in 37°C, 5% CO2 incubator with water‐saturated atmosphere.

### 2.3 Cell culture and transfection

The 293T cells were cultured in complete growth medium DMEM (Hyclone, UT, USA), supplemented with 10% heat‐inactivated foetal bovine serum (10% FBS), penicillin (100 U/mL) and streptomycin (100 μg/mL) at 37°C, 5% CO_2_. The siRNA, mimics or inhibitor was purchased from GenePharma (Shanghai, China). Lipofectamine 2000 Reagent (Invitrogen, CA, USA) was used in all of the transfection experiments following the manufacturer's instructions.

### 2.4 RNA Extraction and qRT‐PCR

Total RNA was isolated from bowel tissue and cell line using the TRIzol reagent according to the manufacturer's protocol (Life Technologies, CA, USA). Then, the OD260, OD280 and OD260/OD280 ratios of total RNA were measured using a Beckman DU‐800 UV spectrophotometer. 500 ng RNA was reverse‐transcribed into cDNA by Reverse Transcription Kit (Takara, Tokyo, Japan) for polymerase chain reaction. The PCR primer sequences for these genes are shown in Table 2 and were synthesized by Realgene (Nanjing, China). PCR amplification was processed as follows: 1 × (95°C, 30 seconds); 40 × (95°C, 5 seconds; 60°C, 30 seconds);1 × (95°C, 15 seconds; 60°C, 60 seconds; 95°C, 15 seconds). All experiments were repeated three times.

**Table 2:** Sequences of primers for qRT‐PCR‐ and siRNA‐related sequence

Name	Sequence	
GAPDH	Forward	5′‐GCACCGTCAAGGCTGAGAAC‐3′
	Reverse	5′‐GGATCTCGCTCCTGGAAGATG‐3′
ADAR1	Forward	5′‐CTGAGACCAAAAGAAACGCAGA‐3′
	Reverse	5′‐GCCATTGTAATGAACAGGTGGTT‐3′
ADAR2	Forward	5′‐GTGAAGGAAAACCGCAATCTGG‐3′
	Reverse	5′‐CAGGAGTGTGTACTGCAAACC‐3′
STAU1	Forward	5′‐TTGTGACCAAGGTTTCGGTTG‐3′
	Reverse	5′‐AGCTATGGCGGCATTTTTCTTT‐3′
mmu‐ADAR2	Forward	5′‐GCGAGGGTATTCCGCTCTC‐3′
	Reverse	5′‐GGTACTTGGAGTGACCATTGCT‐3′
TLR4	Forward	5′‐AGACCTGTCCCTGAACCCTAT‐3′
	Reverse	5′‐CGATGGACTTCTAAACCAGCCA‐3′
mmu‐TLR4	Forward	5′‐GCCTTTCAGGGAATTAAGCTCC‐3′
	Reverse	5′‐GATCAACCGATGGACGTGTAAA‐3′
CCNT2	Forward	5′‐CAGCGTCTCAATGTCTCTCAG‐3′
	Reverse	5′‐GAGTCTGTTGAAGGTAAGCATCA‐3′
U6	Forward	5′‐CTCGCTTCGGCAGCACA‐3′
	Reverse	5′‐AACGCTTCACGAATTTGCGT‐3′
miR‐142‐3p	Forward	5′‐ACACTCCAGCTGGGTGTAGTGTTTCCTACTT‐3′
	Reverse	5′‐CTCAACTGGTGTCGTGGAGTCGGCAATTCAGTTGAGTCCATAAA‐3′
ADAR2 siRNA1	Sense	5′‐CCUGGUCCUGGGUAAGUUUTT‐3′
	Antisense	5′‐AAACUUACCCAGGACCAGGTT‐3′
ADAR2 siRNA2	Sense	5′‐GGAGAAUGUCCAGUUUCAUTT‐3′
	Antisense	5′‐AUGAAACUGGACAUUCUCCTT‐3′
miR‐142 mimics	Sense	5′‐UGUAGUGUUUCCUACUUUAUGGA‐3′

### 2.5 RNA editing site‐specific qPCR (RESSqPCR)

In order to implement a rapid and cost‐effective method to detect an RNA editing fingerprint, we devised an RNA editing site‐specific primer design strategy that is compatible with SYBR green qRT‐PCR protocols (RESSqPCR). We have previously developed qRT‐PCR primers that specifically recognize A‐to‐I editing, and here, we employed a similar approach in designing RESSqPCR primers.^18^

### 2.6 Protein extraction and Western blot analysis

Total protein was extracted from bowel tissue and cell lines by RIPA buffer plus PMSF (100 μL RIPA+1 μL PMSF). Each protein sample contains 80 μL of protein, 5 μL of 5× loading buffer (Beyotime, Nantong, China), and RIPA (Beyotime, Nantong, China) was supplemented to 25 μL which was incubated for 5 minutes at 100°C. Select the appropriate gel density according to the size of the target protein. Add 25 μL of protein sample carefully to each well of gel. The membranes were incubated with the primary antibodies: Rabbit polyclonal ADAR2, STAU1 (ProteinTech, Chicago, IL), GAPDH (Beyotime, Nantong, China) and then incubated with corresponding peroxidase‐conjugated secondary antibody. The expression of GAPDH was used as an internal control.

### 2.7 Cell proliferation

Cells were plated on 96‐well plates and incubation with CCK8 (Beyotime, Nantong, China) for 1 hour; subsequently, the TECAN infinite M200 Multimode microplate reader (Tecan, Mechelen, Belgium) was used to measure the absorbance at 450 nm. Also, the EDU assay was taken to detect the proliferation of cells. The EDU assay used EdU (5‐ethynyl‐2′‐deoxyuridine; Roche) (Ribobio, Guangzhou, China) to stain the proliferating cells. Fluorescence staining was determined using a confocal microscope (Olympus, Tokyo, Japan). All experiments were repeated three times independently.

### 2.8 Immunofluorescence assay

Cells were plated on 96‐well plates. Immunofluorescence staining of cells was handled by 1% Triton X‐100, incubated by primary antibody against TUG1 (1:1000, Abcam) at 4°C overnight. After washing extensively with phosphate‐buffered saline, sections were incubated with secondary antibody conjugated with FITC. Staining results were evaluated under microscope.

### 2.9 Transwell assay

Cells were cultured on 6‐well plates and treated differently. About 100 μL cell suspension with serum‐free medium was seeded in the upper chamber, and 600 μL medium with 10% foetal bovine serum in the lower chamber. After transfected 48 hours, cells were fixed with methanol and then dyed with crystal violet staining solution, counted and photographed under 40× magnification (nine views per well). All experiments were repeated three times independently.

### 2.10 Plasmid constructs

The ADAR2 overexpression plasmid was purchased from GenePharma (Shanghai, China). The binding site of STAU1 was inserted into the KpnI and SacI sites of pGL3 promoter vector (Realgene, Nanjing, China) to get the pGL3‐STAU1‐Wild plasmid, and the binding site was mutated antisense to clone to the pGL3 vector called pGL3‐STAU1‐Mut.

### 2.11 Sanger sequencing

The amplification products were inserted into a T‐vector for Sanger sequencing to detect the A‐to‐I RNA editing. The primers of pri‐miR‐142 were designed: 5′‐TGGAGCAGGAGTCAGGAGG‐3′ (sense) and 5′‐GCCGAGGAAGATGGTGG‐3′ (antisense) and were synthesized by Tsingke (Nanjing, China). The Sanger sequencing was performed by Tsingke too.

### 2.12 ELISA

The concentration of LPS was determined in HSCR and control specimen by ELISA according to the manufacturer's instructions. The concentration was measured by microplate reader on a spectrophotometric enzyme‐linked immunosorbent sandwich assay (ELISA) plate, and the reading was measured at 450 nm. The standard curve was drawn according to the test results of the standard, and the concentration of each sample was calculated.

### 2.13 Dual‐luciferase reporter assay

The miR‐142‐3p mimics and pGL3‐STAU1 wild‐type plasmids, miR‐142‐3p mimics and pGL3‐STAU1 mutant plasmids, Negative control (NC) and pGL3‐AKT3 wild‐type plasmid, NC and pGL3‐AKT3 mutant plasmids (Realgene, Nanjing, China) were transfected with Renilla luciferase vector pRL‐SV40 (5 ng) into 293T cells plated on 24‐well plates using lipofectamine 2000 (Invitrogen, Shanghai, China). The Renilla luciferase vector pRL‐SV40 was also used to normalize the differences in transfection efficiency. After incubation for 48 hours, the cells were treated according to manufacturer's protocol using the Dual‐Luciferase Assay (Promega, Madison, WI, USA). Transfection was repeated three times in triplicate.

### 2.14 Statistical analysis

The experiments were performed in triplicate independently. Chi‐square tests and Student's *t* test were used to evaluate statistical differences in demographic and clinical characteristics. Data were considered statistically significant as follows: *P <* *.05, **P <* *.01 and ***P <* *.001.

## 3 RESULT

### 3.1 LPS inhibits the cell proliferation and differentiation

**Figure 1:**
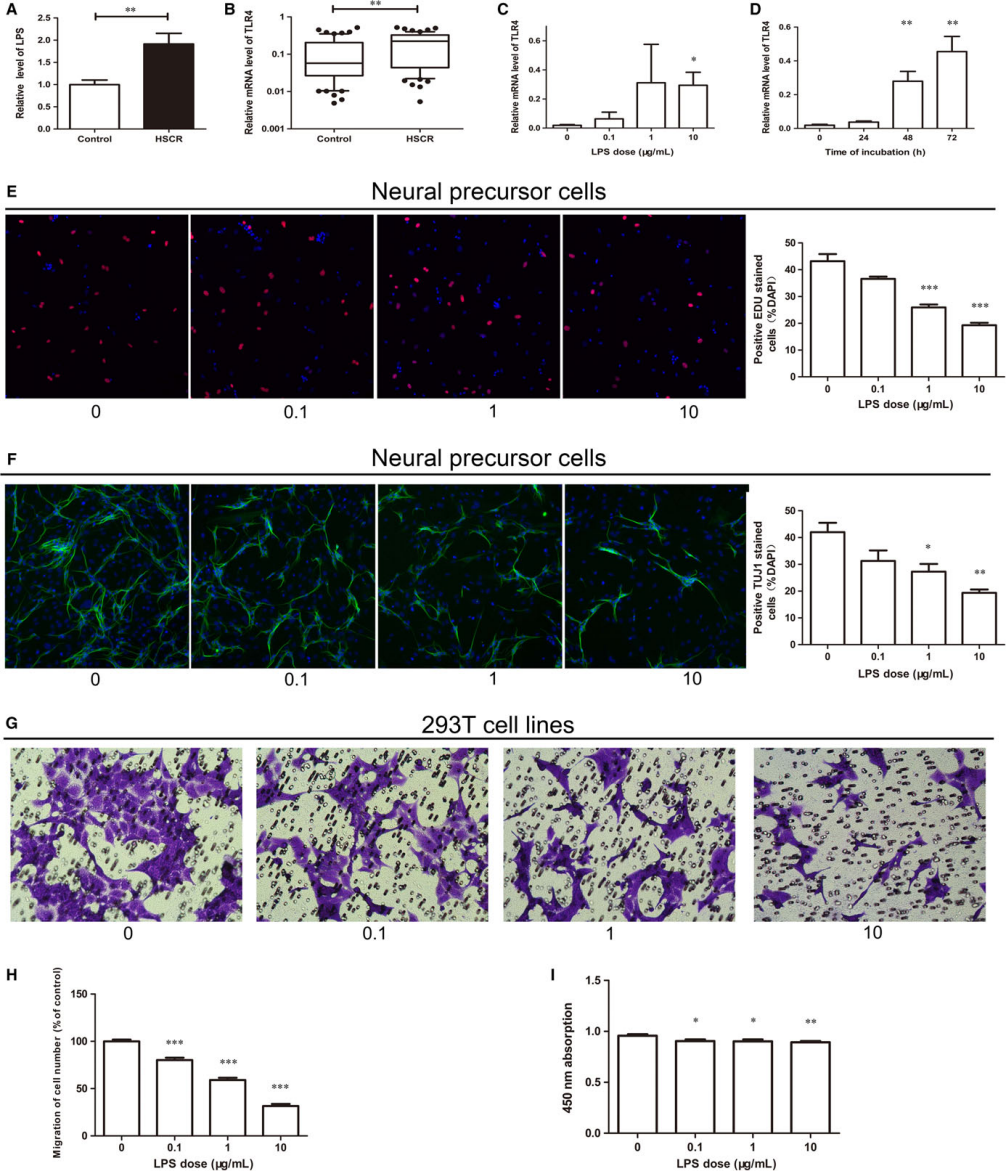
Cytobiology change after treating cells with LPS A, The LPS level in HSCR tissues and control tissues was detected by ELISA. B, qRT‐PCR analysis of TLR4 expression in control tissues and HSCR tissues. C and D, qRT‐PCR was performed to exam the TLR4 level of neural precursor cells (NPC) treated with LPS in a time‐ and dose‐dependent manner. E, Proliferation of NPC treated with LPS at dose of 0, 0.1, 1, 10 μg/mL for 48 h was determined by EDU. F, Differentiation of NPC was assessed by immunofluorescence. G and H, The Transwell assay was carried out in 293T cell line to measure the migration capacity of cells treated with LPS. I, CCK8 assay was carried out to detect the proliferative capacity of LPS manipulated cells in 293T cell line. *indicates significant difference (*P* < 0.05). **indicates remarkable difference (*P* < 0.01). ***indicates statistical significant differences at *P* < 0.001.

Previous studies suggest that the lipopolysaccharide (LPS)^19^, ^20^ has a function of neuroprotection and can regulate the GI motility. Initially, the concentration of LPS was detected in intestinal tissue, and the relative LPS concentration of HSCR segment was higher than control segment as shown in Figure 1A. Then, we tested the mRNA level of TLR4, the receptor of LPS, in HSCR and control samples. TLR4 was overexpressed in colon tissues from HSCR (Figure 1B), and these results indicate that LPS may be involved in HSCR pathology. Different concentrations of LPS (0, 0.1, 1, 10 μg/mL) for 72 hours, or concentration of 10 μg/mL at different time‐points (24, 48, 72 hours) was added to neural precursor cells (NPC). The mRNA level of TLR4 was used to observe the effects of LPS (Figure 1C,D). After treatment, the proliferation and differentiation of NPC are shown in Figure 1E,F. The ratio of proliferation and differentiation was decreased most obviously at final concentration of 10 μg/mL of 72 hours LPS treatment. The CCK8 and Transwell assay were made over the 293T cells to show the suppression of proliferation and migration (Figure 1G‐I).

### 3.2 LPS increases ADAR2 expression in cells

**Figure 2:**
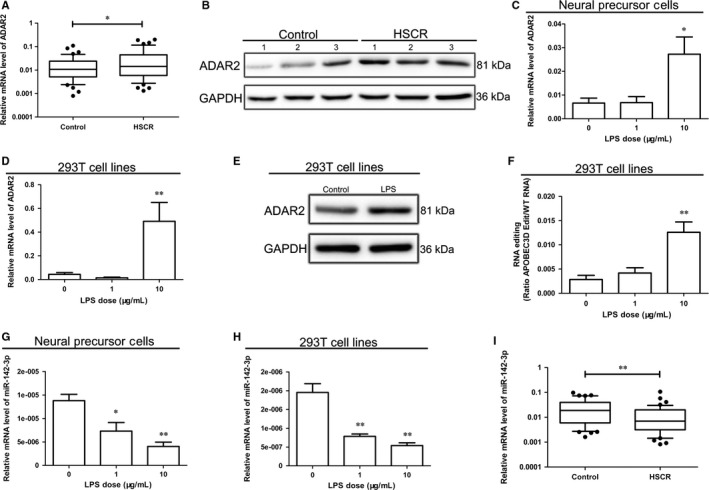
ADAR2 and miR‐142‐3p expressions in crowd sample and cells treated by LPS A and B, mRNA and protein expression levels of ADAR2 in control samples and HSCR samples. C and D, mRNA expression of ADAR2 in NPCs and 293T cells co‐transduced with LPS. E, Protein level of ADAR2 in 293T cells cultured with 10 μg/mL LPS. F, RESSqPCR analysis of RNA editing ratio of APOBEC3D in 293T cells transduced with different dose of LPS (0, 1, 10 μg/mL). G and H, mRNA expression of miR‐142‐3p in NPCs and 293T cells incubated with LPS. I, mRNA expression of miR‐142‐3p in control tissues and HSCR tissues. *indicates significant difference (*P* < 0.05). **indicates remarkable difference (*P* < 0.01).

As the LPS dramatically causes rise to ADAR1 editing activity and promotes ADAR1‐mediated A‐to‐I editing as demonstrated before,^11^ we first tested the ADAR1 expression in HSCR and control tissues, out of our expectation,there was no significant statistical difference (Figure S1A). But still, A‐to‐I editing could also regulate by ADAR2, which belongs to ADAR enzymes. We have been suggested that LPS contributed to activation of ADAR2‐mediated A‐to‐I editing. Then, we detected the expression of ADAR2 in control and HSCR bowel tissues, and the result showed that ADAR2 increased in HSCR tissues (Figure 2A,B). Our study suggested that ADAR2 expression enhanced NPC and 293T cells treated with LPS at different concentrations (0, 1, 10 μg/mL) (Figure 2C,D). The protein level of ADAR2 also increased in 293T cells treated with LPS at a concentration of 10 μg/mL (Figure 2E). Furthermore, the ADAR2′s editing activity measured by RNA editing site‐specific qPCR (RESSqPCR) is shown in Figure 2F.^18^ In summary, these data suggest that LPS strengthens ADAR2‐mediated A‐to‐I editing.

### 3.3 LPS regulates miR‐142‐3p biogenesis

Emerging studies have demonstrated that ADAR2 can impair miRNA biogenesis, leading to tumorigenesis.^21^, ^22^ MiR‐21‐3p, miR‐23b, miR‐383, miR‐99a‐3p, miR‐142‐3p and miR‐142‐5p were selected when integrating the 91 miRNAs^21^ significantly changes in statistics between ADAR2 and ADAR2 E/A which were inverted in the siADAR2 cell line with the 78 miRNA changes in HSCR (Table S1). The ADAR2 mediating A‐to‐I editing usually suppresses the miRNA expression. We have been suggested that these miRNAs may also be disrupted by LPS for the LPS‐activated ADAR2. Then, the mature miRNA expression was tested in NPC and 293T cells, and the data demonstrated that LPS addition inhibited the miR‐142‐3p (Figure 2G,H), while the remaining 5 miRNAs did not change in meeting the requirement (Figure S1B,C). However, the pri‐miR‐142 expression is not changed after LPS treatment in NPC cells or 293T cells (Figure S2A). Also, the miR‐142‐3p was affirmed to be decreased in HSCR tissues (Figure 2I).

### 3.4 ADAR2 edits the pri‐miR‐142‐3p and impairs cell proliferation and migration

**Figure 3:**
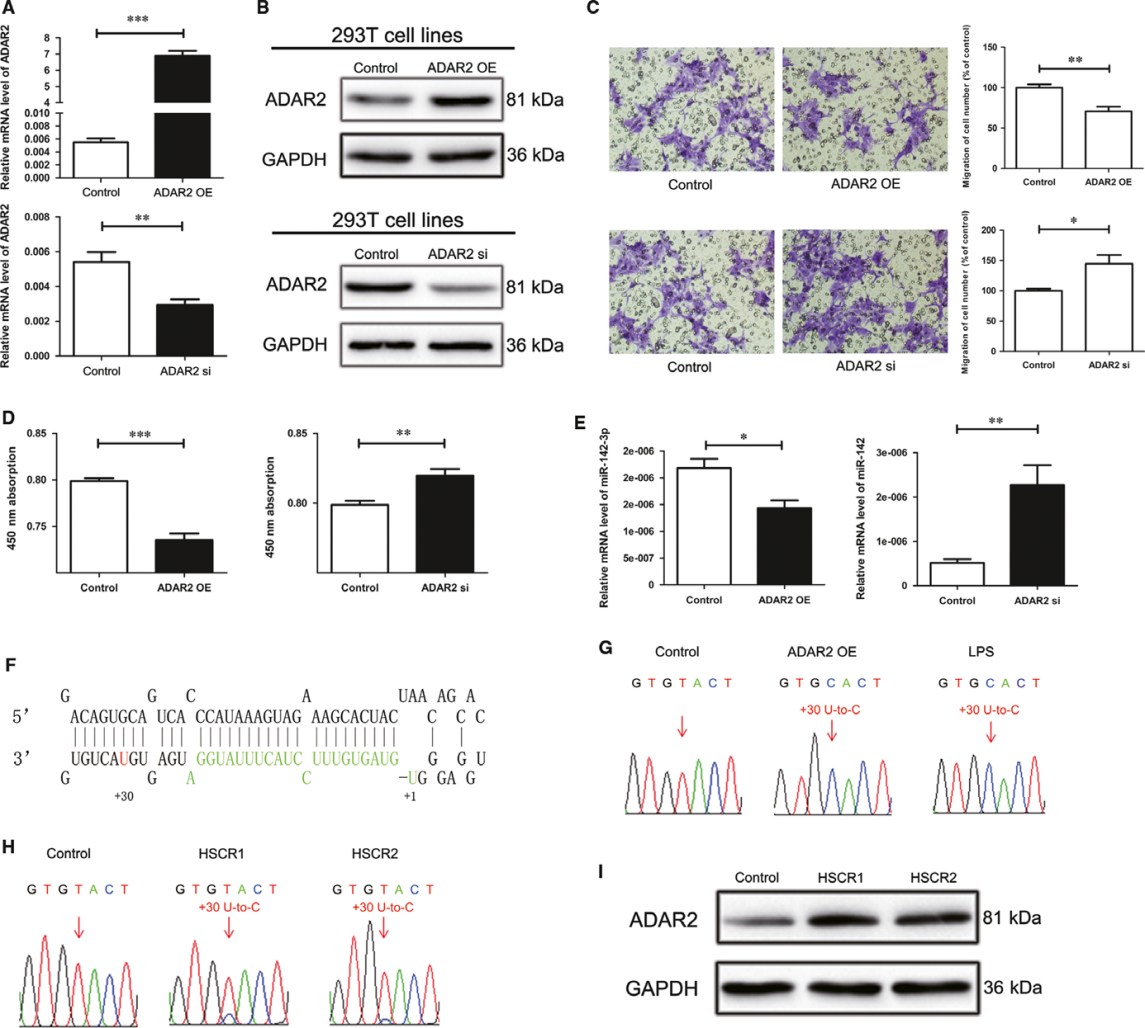
ADAR2 edits the pri‐miR‐142‐3p and impairs cell proliferation and migration A and B, mRNA and protein expression levels of ADAR2 in 293T cells transfected by ADAR2 overexpression vector (upper) or ADAR2 siRNA (lower). C, Transwell assays in 293T cell line treated with ADAR2 overexpression vector (upper) or ADAR2 siRNA (lower). D, The proliferation of ADAR2 overexpression vector (left) or ADAR2 siRNA (right) transfected cells. E, mRNA expression levels of miR‐142‐3p in 293T cells transfected by ADAR2 overexpression vector (left) or ADAR2 siRNA (right). F, The stem‐loop structure for pre‐miR‐142, the nucleotides corresponding to mature miRNA marker in green. The nucleotide positions changed by ADAR2 overexpression were labelled with red. G, Sequencing analysis of 293T cells transfected with the ADAR2 overexpression plasmid or LPS at dose of 10 μg/mL. The nucleotide residues that display the editing events are marked with red arrows. H, The sequencing results of miR‐142‐3p in 20 control tissues and 20 HSCR tissues. The nucleotide residues changes were labelled with red arrows. The number above the arrows indicates the positions relative to that of the mature miRNAs. I, The protein expression of ADAR2 in the two HSCR tissues was examined by Western blot. *indicates significant difference (*P* < 0.05). **indicates remarkable difference (*P* < 0.01). ***indicates statistical significant differences at *P* < 0.001.

To determine whether ADAR2 is required for cell function, the gain and loss function of ADAR2 was carried out in 293T cells to test the proliferation and migration. The ADAR2 was increased by plasmid (Figure 3A,B) with dramatical repression of cell proliferation and migration ability compared with control (Figure 3C,D). To investigate the association between ADAR2 and miR‐142‐3p, we used cells transfected with overexpression vector, confirming that active ADAR2 significantly reduced the level of mature miR‐142‐3p (Figure 3E). Conversely, down‐regulation of ADAR2 in 293T cells increased mature miR‐142‐3p expression (Figure 3E). In addition, the numbers of proliferous and migrated cells were significantly enhanced in cells when ADAR2 was suppressed by ADAR2 siRNA (Figure 3C,D).

The qRT‐PCR products from the post‐transfection cells were processed to detect A‐to‐G changes editing events in the cDNA sequences of pri‐miR‐142. The qRT‐PCR products from ADAR2 overexpressing vector‐infected cells were collected for TA‐cloning; 50 clones were selected for sequencing subsequently. Figure 3F illustrates the sites that nucleotide changes when comparing with the sequence of samples treated with negative control relative to the mature miR‐142‐3p. This change did not appear in cells transfected with negative control but in the cells overexpressing ADAR2 or at a high concentration of LPS (Figure 3G), which favour the editing events regulated by ADAR2. Nucleotide changes of miR‐142 precursors evaluate in the 293T cells overexpressing ADRA2 were U‐to‐C changes because ADAR2‐mediated A‐to‐I editing may occur on RNA transcripts complementary to pri‐miRNAs.^22^

### 3.5 ADAR2 is associated with the nucleotide changes in pri‐miRNAs in HSCR samples

Then, we tested whether the clinical specimens also appeared the ADAR2‐mediated nucleotide changes in 293T cells. The qRT‐PCR products of cDNA from 20 HSCR and control samples were sequenced to detect the editing sites of pri‐miR‐142‐3p. Two HSCR samples showed a U‐to‐C change at the sequence of pri‐miR‐142‐3p (Figure 3H) and such change did not occur on that of the control tissues. Interestingly, the editing event occurred on HSCR tissues seemed in accordance with the nucleotide change appeared in 293T cells overexpressed ADAR2. As our experiments suggested that A‐to‐G editing could be induced by ADAR2, we would have to observe whether ADAR2 was enhanced in HSCR tissues which have been detected by nucleotide change. The results demonstrated that the ADAR2 in HSCR was significantly higher than that in the control group (Figure 3I). All these results suggested that HSCR was associated with ADAR2‐mediated A‐to‐I editing caused by ADAR2 elevation.

### 3.6 ADAR2‐mediated editing on pri‐miR‐142‐3p functionally affects the target gene of miR‐142‐3p

**Figure 4:**
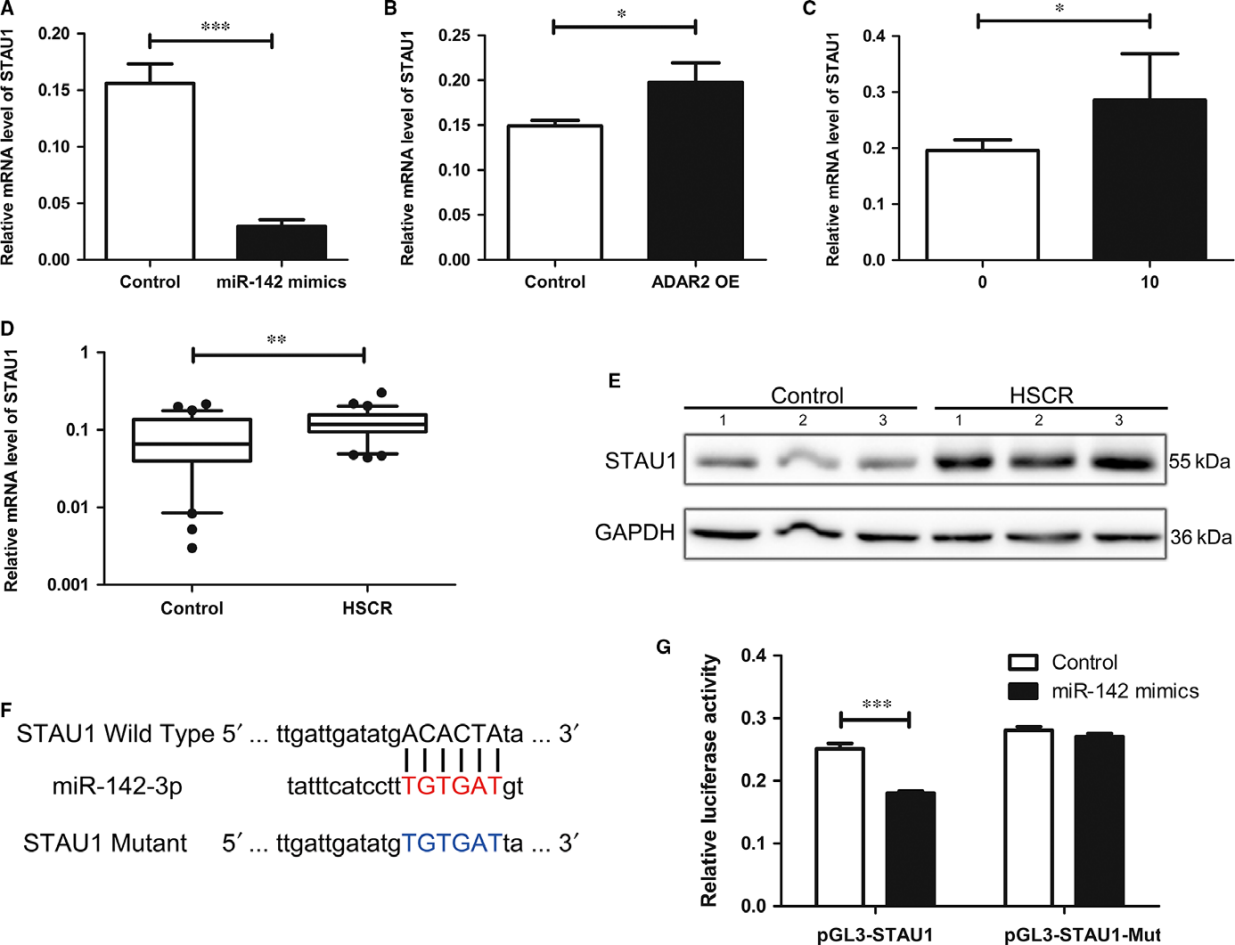
ADAR2‐mediated editing on the pri‐miR‐142‐3p functionally affects the STAU1 A‐C, mRNA expression levels of STAU1 in 293T cells transfected by miR‐142 mimics (A) or ADAR2 overexpression vector (B) or 10 μg/mL LPS (C). D, Relative expression of STAU1 in HSCR tissues in comparison with control tissues. E, Protein level of STAU1 in HSCR tissues and normal control samples was detected by Western blot. F, The putative miRNAs binding sites was cloned downstream of the vector and named pGL3‐STAU1‐wild. Bottom: Mutations in the STAU1 sequence to create the mutant luciferase reporter constructs named pGL3‐STAU1‐Mut. G, The luciferase reporter in 293T cell line. *indicates significant difference (*P* < 0.05). **indicates remarkable difference (*P* < 0.01). ***indicates statistical significant differences at *P* < 0.001.

To further study the impact of the editing event mediated by ADAR2 on miR‐142‐3p targets, the bioinformatic prediction (DIANA, miRanda, PicTar, PITA, TargetScan) was used to point out *RAC1, STAU1, CCNT2* as putative miR‐142‐3p target genes. MiR‐142‐3p mimics were transfected to 293T cells, which lead to a reduction in the level of STAU1 (Figure 4A), but not RAC1 and CCNT2 (Figure S1D,E). Moreover, overexpression of ADAR2 or adding LPS at the concentration of 10 μg/mL to 293T cells also caused an increase in STAU1 (Figure 4B,C), which demonstrated that the ADAR2‐mediated editing event affects miR‐142‐3p target gene. In clinical specimens, STAU1 mRNA level was obviously increased in HSCR patients in contrast to control patients. The protein level of STAU1 was in parallel with the mRNA levels (Figure 4D,E).

To determine whether miR‐142‐3p acts on STAU1, the 293T cells were transfected with plasmid pGL3‐STAU1 and pGL3‐STAU1‐Mut, which contains the predicted binding site and mutant binding site (Figure 4F). The luciferase reporter assay elucidated that miR‐142‐3p mimics inhibited luciferase activity in pGL3‐STAU1‐transfected cells. Meanwhile, there were no changes in the luciferase activity of pGL3‐STAU1‐Mut‐transfected cells between miR‐142‐3p mimics and control group (Figure 4G). In general, our findings proved that STAU1 is a direct target gene of miR‐142‐3p. The STAU1 was decreased by STAU1 siRNA and increased by STAU1 plasmid both in mRNA level and in protein level (Figure S2C,D). The Transwell and CCK8 assay showed that STAU1 overexpression decreased the ability of cells to proliferate and migrate while the ability was inhibited by STAU1 siRNA (Figure S2E,F).

### 3.7 LPS‐ADAR2‐miR‐142‐3p is critical for cellular functions

**Figure 5:**
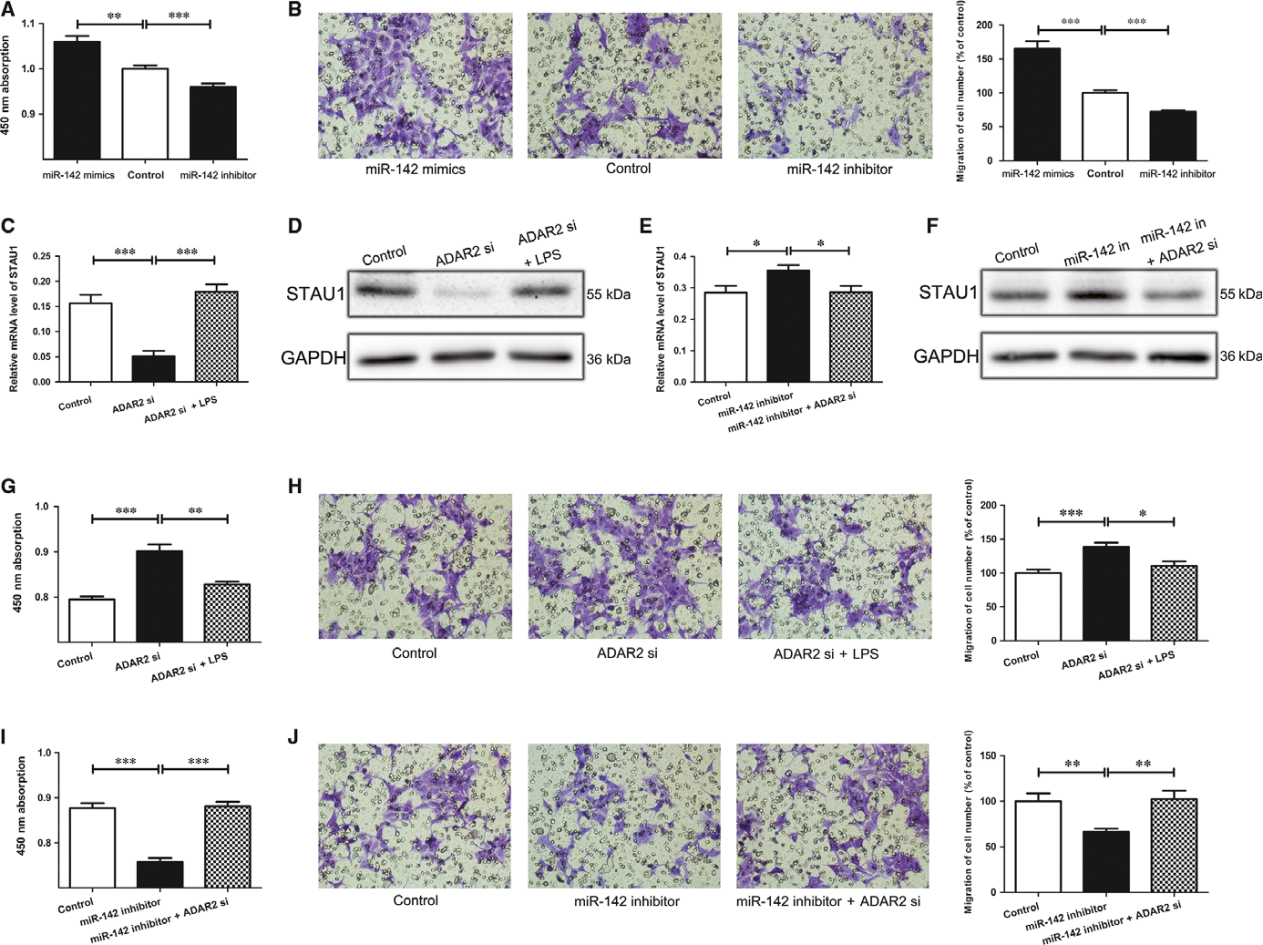
LPS‐ADAR2‐miR‐142‐3p is critical for cell functions A, Cell proliferation of 293T cell lines transfected with miR‐142‐3p mimics and inhibitor. B, Cell migration was detected using the Transwell assays. C and D, ADAR2 siRNA with or without LPS was transfected into 293T cells; the mRNA level (C) and protein level (D) of STAU1 were evaluated by qRT‐PCR(C) and Western blot, respectively (D). E, 293T cells were transfected with miR‐142‐3p inhibitor with or without ADAR2 siRNA and qRT‐PCR was used to detect the relative mRNA levels of STAU1. F, Relative protein level of STAU1 in 293T cells when transfected with miR‐142 inhibitor or miR‐142 inhibitor plus ADAR2 siRNA. G and H, The proliferation and migration ability of 293T cells were detected by CCK8 and Transwell assays after treated with ADAR2 siRNA with or without LPS. I and J, CCK8 assay and Transwell assays were performed to detect the proliferation and migration of cells transfected by miR‐142 inhibitor and treated with miR‐142 inhibitor plus ADAR2 siRNA. *indicates significant difference (*P* < 0.05). **indicates remarkable difference (*P* < 0.01). ***indicates statistical significant differences at *P* < 0.001.

We next checked whether miR‐142‐3p participated in cell migration or proliferation. MiR‐142‐3p mimics and inhibitor were added to 293T cells to observe the migration and proliferation. The Transwell and CCK8 assay showed that miR‐142‐3p overexpression enhanced the number of proliferative or migratory cells (Figure 5A,B). In contrast, the cell proliferation or migration ability could be reversed by miR‐142‐3p inhibitor, which was consistent with the cells treated by LPS and ADAR2 plasmid (Figure 5A,B). However, the accelerative effect of ADAR2 siRNA on cell proliferation or migration was reversed by LPS (Figure 5G,H) and so was the STAU1 level (Figure 5C,D). Meanwhile, the expression of STAU1 promoted by miR‐142‐3p inhibitor was inverted by ADAR2 siRNA (Figure 5E,F) and ADAR2 siRNA could partially abrogate the depression effect on cell proliferation and migration of miR‐142‐3p inhibitor (Figure 5I,J).Yet, we established a connection between LPS, ADAR2, pri‐miR‐142, miR‐142‐3p and STAU1 in HSCR progression.

## 4 DISCUSSION

Lipopolysaccharide has emerged as the main driving force of gastrointestinal motility^8^ and could damage the enteric neuron of rat or porcine.^9^, ^10^ The dysplasia of ENS is responsible for many disorders including HSCR. Moreover, a recent report described that the A‐to‐I editing events conferred by ADAR activation was enhanced by LPS.^11^ Further more,another previous study reported that gain or loss of ADAR contributes to cancer progression by regulating the maturity of several miRNAs modulated by RNA editing.^23^ Taken together, our study provides a novel link between LPS‐ and ADAR2‐mediated miRNA maturity in HSCR.

Our results showed that high concentration of LPS inhibited the proliferation and differentiation of NPC and impeded the migration of 293T cells, while some researchers showed that low‐dose LPS induced neuroprotection.^20^ It seems that the LPS affects the NPC in a dose‐dependent manner. Significantly, enhanced ADAR1 activity was observed in LPS‐stimulated alveolar macrophages.^24^ But in our study, ADAR1 activity was not affected by LPS and we found another ADAR enzyme; ADAR2 was activated by increased LPS signal in cells. Also, the A‐to‐I editing activity was invoked detected by RNA editing site‐specific qPCR (RESSqPCR).

Recently, researchers have established relationship between ADAR‐mediated RNA editing and gastric cancer^25^ or liver cancer^22^ pathogenesis and progression. In our research, the ADAR2 was overexpressed and inhibited in 293T to verify the proliferation and migration. To investigate whether ADAR‐mediated RNA editing contributes to the abnormal expression of miRNAs in HSCR, we got intersection elements from the miRNAs changing in HSCR and cell treated with ADAR2 siRNA^21^; six miRNAs were found possibly edited by ADAR2 in HSCR. The expression of miR‐142‐3p was increased when silence ADAR2 and the expression reversed when elevating ADAR2. Previously, A‐to‐I editing events have been reported to occur in several miRNA precursors.^26^ The HSCR samples were sequenced to detect whether A‐to‐I editing in maturity of pri‐miR‐142‐3p was associated with HSCR. However, in our study, it is clear that the overexpression of ADAR2 and the high concentration of LPS can regulate such A‐to‐I editing in accordance with that in HSCR samples. Our data confirmed that the miR‐142‐3p may be edited by ADAR2 and the sequencing data revealed an additional editing site only in 293T cells. It was better if the NPC was treated and the A‐to‐I editing was also detected.

According to previous research, miR‐142‐3p is involved in cell migration, proliferation and apoptosis in renal cell carcinoma.^27^ Moreover, lower expression of miR‐142‐3p inhibited the cell migration and proliferation, which was just consistent with the representation of overexpression of ADAR2. Predicted target genes for miR‐142‐3p from DIANA, miRanda, PicTar, PITA and TargetScan identified 3 genes (*RAC1, STAU1, and CCNT2*) as potential targets for miR‐142‐3p. Only STAU1 was inhibited by miR‐142‐3p mimics. STAU1 encodes a protein that forms granules initially thought to be neuronal active macromolecular structures,^27^ and in colorectal cancer, the expression of Staufen gene was significantly lower than that in corresponding distal normal tissue at mRNA level.^28^ When LPS was in high concentration and ADAR2 was highly expressed, STAU1 levels increased, most probably owing to the suppression of miR‐142‐3p. Thus, miR‐142‐3p accelerated cell migration by targeting STAU1.

The key progress of this research is that ADAR2 editase activity impairs miR‐142‐3p biogenesis and decreased cell migration capacity, resulting in malignant reprogramming to HSCR pathogenesis. This overexpression of ADAR2 gave rise to A‐to‐I RNA editing in 293T cell lines, particularly in miR‐142‐3p. In addition, we showed that enhanced LPS signal results in ADAR2 activation. Later on, miR‐142‐3p enhanced in control tissues and more of the edited form accumulated in HSCR tissues. MiR‐142‐3p accelerated migration and proliferation of cells via down‐regulation of the STAU1. In summary, we established a pivotal LPS‐ADAR2‐miR‐142‐3p‐STAU1 axis to provide a link between LPS and HSCR.

## CONFLICTS OF INTEREST

None.

## AUTHOR CONTRIBUTION

Weibing Tang, Yankai Xia, Xiaoqun Xu and Lei Peng designed the experiments; Lei Peng and Yang Su performed the experiments; Hua Xie, Hongxing Li, Chunxia Du,Ziyang Shen,Weiwei Jiang and Jin Yan analysed the data; Lei Peng, Hua Zhang and Qiyang Shen wrote the manuscript. All authors discussed the results and commented on the manuscript.
