# Corrigendum

**DOI:** 10.1111/jcmm.14106

**Published:** 2019-01-28

In Peng et al,^1^ the published article contains errors in Figures 3C and 5H. The correct figures are shown below. The authors confirm all results and conclusions of this article remain unchanged. The online version has been corrected.

**Figure 3:**
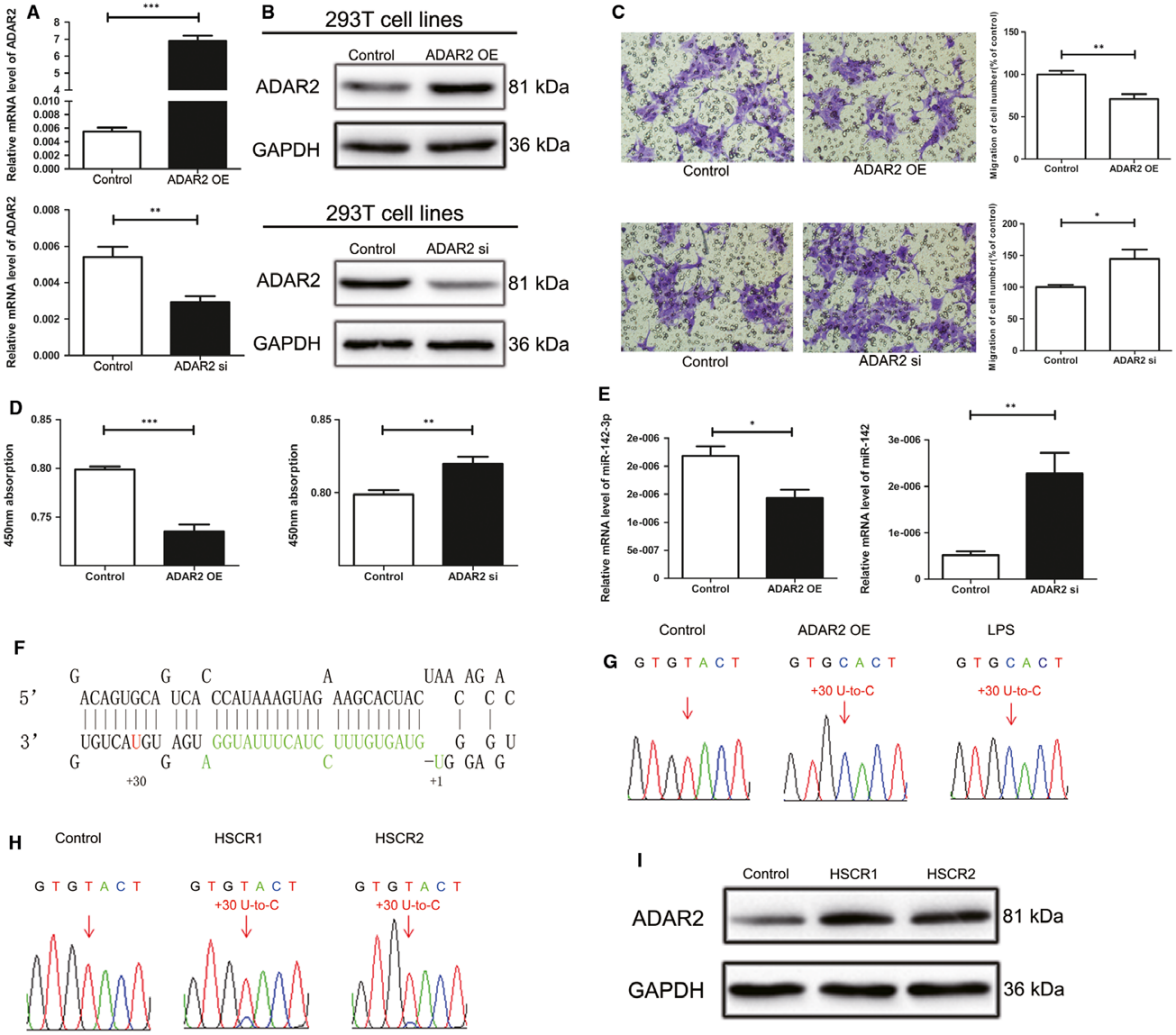
ADAR2 edits the pri‐miR‐142‐3p and impairs cell proliferation and migration A and B, mRNA and protein expression levels of ADAR2 in 293T cells transfected by ADAR2 overexpression vector (upper) or ADAR2 siRNA (lower). C, Transwell assays in 293T cell line treated with ADAR2 overexpression vector (upper) or ADAR2 siRNA (lower). D, The proliferation of ADAR2 overexpression vector (left) or ADAR2 siRNA (right) transfected cells. E, mRNA expression levels of miR‐142‐3p in 293T cells transfected by ADAR2 overexpression vector (left) or ADAR2 siRNA (right). F, The stem‐loop structure for pre‐miR‐142, the nucleotides corresponding to mature miRNA marker in green. The nucleotide positions changed by ADAR2 overexpression were labelled with red. G, Sequencing analysis of 293T cells transfected with the ADAR2 overexpression plasmid or LPS at dose of 10 lg/mL. The nucleotide residues that display the editing events are marked with red arrows. H, The sequencing results of miR‐142‐3p in 20 control tissues and 20 HSCR tissues. The nucleotide residues changes were labelled with red arrows. The number above the arrows indicates the positions relative to that of the mature miRNAs. I, The protein expression of ADAR2 in the two HSCR tissues was examined by Western blot. *indicates significant difference (*P* < 0.05). **indicates remarkable difference (*P* < 0.01). ***indicates statistical significant differences at *P* < 0.001.

**Figure 5:**
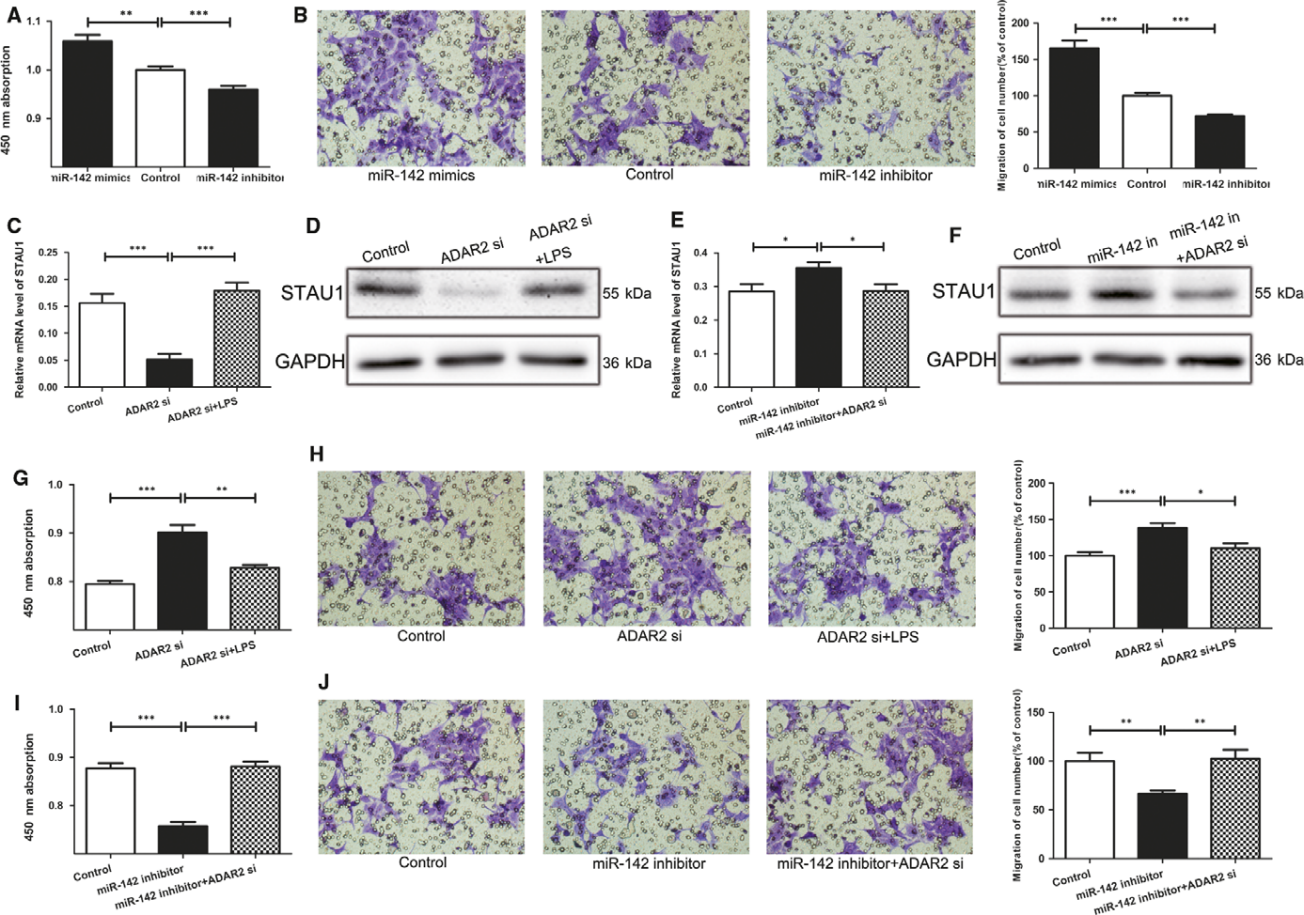
LPS‐ADAR2‐miR‐142‐3p is critical for cell functions A, Cell proliferation of 293T cell lines transfected with miR‐142‐3p mimics and inhibitor. B, Cell migration was detected using the Transwell assays. C and D, ADAR2 siRNA with or without LPS was transfected into 293T cells; the mRNA level (C) and protein level (D) of STAU1 were evaluated by qRT‐PCR(C) and Western blot, respectively (D). E, 293T cells were transfected with miR‐142‐3p inhibitor with or without ADAR2 siRNA and qRT‐PCR was used to detect the relative mRNA levels of STAU1. F, Relative protein level of STAU1 in 293T cells when transfected with miR‐142 inhibitor or miR‐142 inhibitor plus ADAR2 siRNA. G and H, The proliferation and migration ability of 293T cells were detected by CCK8 and Transwell assays after treated with ADAR2 siRNA with or without LPS. I and J, CCK8 assay and Transwell assays were performed to detect the proliferation and migration of cells transfected by miR‐142 inhibitor and treated with miR‐142 inhibitor plus ADAR2 siRNA. *indicates significant difference (*P* < 0.05). **indicates remarkable difference (*P* < 0.01). ***indicates statistical significant differences at *P* < 0.001.

The authors apologize for the inconvenience this may cause.
