# Supplementary figures and images for: Lipopolysaccharide enhances ADAR2 which drives Hirschsprung's disease by impairing miR‐142‐3p biogenesis
Source: J Cell Mol Med. 2018 Jun 29;22(9):4045–55. doi: 10.1111/jcmm.13652 (PMC6111854; doi:10.1111/jcmm.13652)

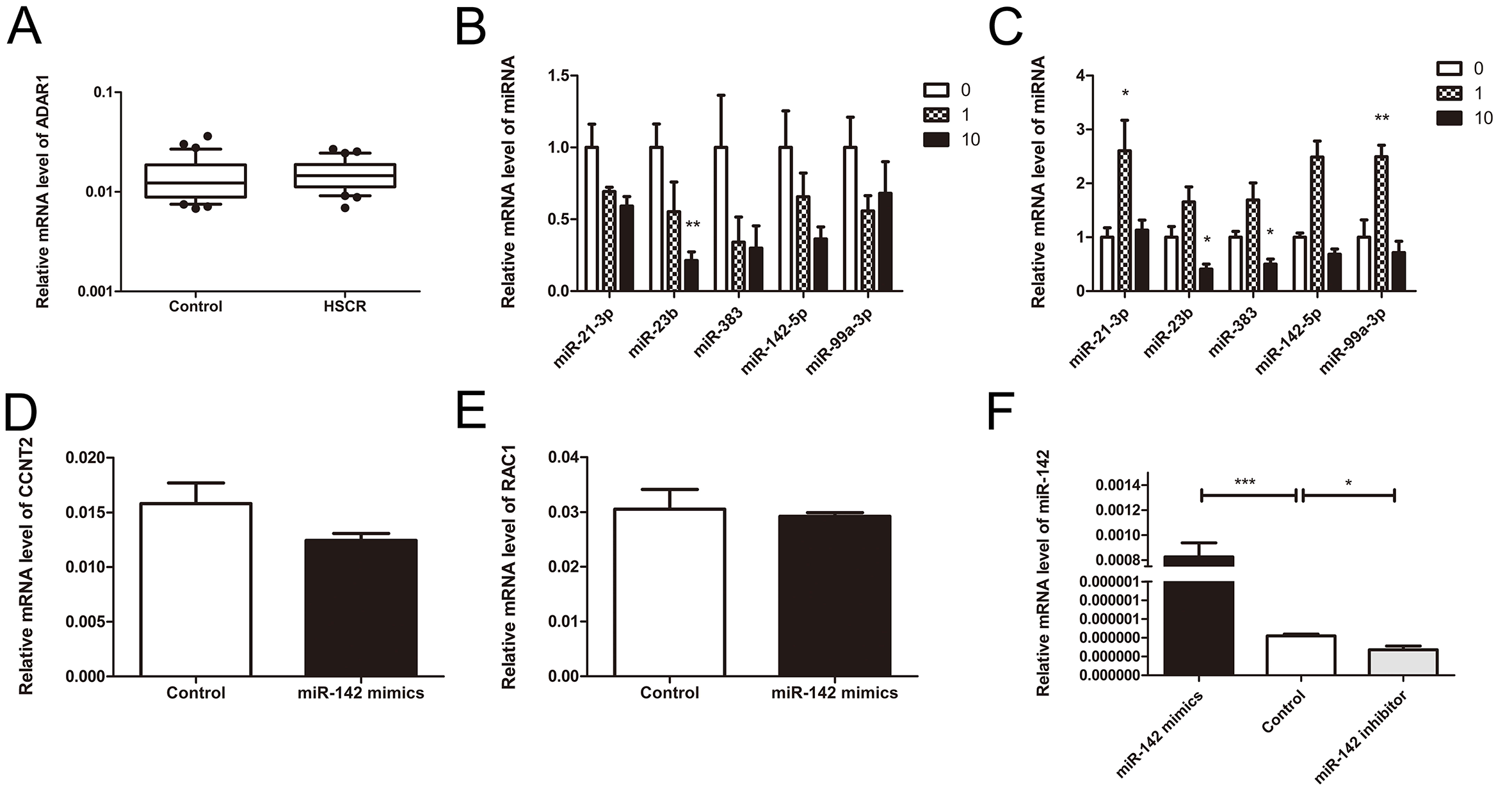

Supplement: Supplementary file 1 [file JCMM-22-4045-s001.tif]
